# The Berrettini Anastomosis’ Morphology and “Probable Zone”: A Cadaveric Study

**DOI:** 10.7759/cureus.78967

**Published:** 2025-02-13

**Authors:** Mathew Mendoza, Albert Sarpong, Dallas Bennett, Madeline Ayala, Paul Tran, Chakravarthy Sadacharan, Samantha P Tippen

**Affiliations:** 1 Department of Biomedical Sciences, Tilman J. Fertitta Family College of Medicine, Houston, USA; 2 Department of Anatomy, Baylor College of Medicine, Houston, USA

**Keywords:** anatomical variations, berrettini anastomosis, median nerve, palmar neural connections, ulnar nerve

## Abstract

Introduction

Neural anastomoses between the median nerve (MN) and ulnar nerve (UN), including the Berrettini anastomosis (BA), influence the sensory innervation of the hand. Although typically asymptomatic, the BA's anatomical variability can have clinical implications, particularly during surgical procedures. This study aimed to investigate the prevalence, anatomical variations, and probable location of the BA in cadaveric hands.

Methods

A total of 104 fresh Caucasian cadaveric hands (52 donors: 26 males, 26 females) were dissected at the Tilman J. Fertitta Family College of Medicine and Baylor College of Medicine, Houston, TX. The dissections focused on the BA's origin, insertion, and branching patterns. Measurements were taken using vertical and horizontal reference points to map its "probable zone." Data were collected by multiple researchers to ensure accuracy, and discrepancies were resolved by averaging measurements. Prevalence and variations were categorized into five distinct types, with detailed illustrations provided.

Results

The BA was identified in 80% (83/104) of hands. Type I, with a single connection from the UN to the MN, was the most common (38%). Other patterns included bifurcating (type II), dual origin (type III), reverse (type IV), and complex fibrous networks (type V). The average BA length was 22.03 ± 7.68 mm, with its "probable zone" spanning 26.11-44.21% of hand width and 36.63-50.21% of hand length.

Conclusion

The BA is a common neural connection with significant anatomical variability. Identifying its "probable zone" provides valuable guidance for surgical procedures, minimizing the risk of sensory deficits in the hand. Further studies are recommended to expand upon these findings.

## Introduction

There are four commonly described anomalous neural connections between the median nerve (MN) and ulnar nerve (UN) in the upper limb, which include the Marinacci anastomosis, Martin-Gruber anastomosis, Riche-Cannieu anastomosis, and Berrettini anastomosis (BA) [1-5]. These neural anastomoses represent axonal cross-connections that can alter the sensory and motor innervation patterns of the upper limb, particularly affecting the intrinsic muscles of the hand [2,5]. Although usually asymptomatic, these variant neural connections may become clinically relevant, manifesting as atypical motor or sensory deficits following traumatic or iatrogenic injury to either the MN or UN [1,2,5-7].

In recent years, comprehensive studies and reviews have provided refined insights into the morphological and anatomical characteristics of these neural anastomoses. A 2015 meta-analysis by Roy et al. identified the BA as the most prevalent neural connection in the upper limb, with a pooled prevalence of 60.9%, which aligns with previously reported rates as high as 94% in a study by Don Griot et al. [2,8]. These high prevalence rates have led to a consensus that the BA should be considered a common neural connection rather than an anatomical variation [2-5,7,9].

BA is classically described as a connection between the third common palmar digital nerve (CDN3) from the MN and the fourth common palmar digital nerve (CDN4) from the superficial branch of the UN in the palm, though variations in its exact origin and insertion have been reported [2-5]. Initially illustrated in 1741 by Pietro Berrettini da Cortona as the ramus communicans cum nervi ulnari, the connection was later formally termed the BA in 1991 by Meals and Calkins [1,2,4-6,9-11]. Various studies have since categorized the BA into different pattern types, with the most common variant described as a palmar sensory communicating branch following an oblique trajectory from the UN to the MN [2-4,6,9].

There is general agreement that the BA is purely sensory in function, innervating the ulnar half of the middle finger and the radial half of the ring finger [2-6,12]. Due to its proximity to the transverse carpal ligament in the superficial palm, the BA is vulnerable to iatrogenic injury during common surgical procedures involving the hand, such as carpal tunnel release, neurovascular island flaps, and Dupuytren’s fasciectomy [1]. Injuries to the BA may result in diminished sensation or hyperesthesia in the region between the third and fourth digits [1-6,9]. Consequently, thorough anatomical knowledge of the BA is essential for accurate preoperative localization and intraoperative identification to minimize the risk of nerve damage [1].

Despite advancements in the understanding and recognition of BA, additional contemporary cadaveric studies are warranted to further corroborate existing data. Therefore, the objective of this cadaveric pilot study is to explore the morphological and anatomical characteristics of the BA and to identify a probable zone within the palm where the BA is most commonly encountered.

## Materials and methods

A total of 104 fresh Caucasian cadaveric hands were meticulously dissected at the Tilman J. Fertitta Family College of Medicine (TJFFCOM), University of Houston and Baylor College of Medicine (BCM) Anatomy Laboratories to evaluate the prevalence and anatomical variations of significant anastomoses between the MN and UN. The procurement and preservation of cadavers adhered to the ethical guidelines set forth by the Texas State Anatomical Board, the TJFFCOM Ethics Committee, and Baylor College of Medicine's Anatomy Education Core and Willed Body Program.

Dissections were conducted across three distinct intervals over a six-month period. Cadaveric specimens were obtained from 52 donors (26 males, 26 females), with an average age at death ranging from 68 to 108 years.

Fine dissection techniques were employed, emphasizing the preservation of wrist structures, including the carpal tunnel, while exposing the superficial communicating branches between the MN and UN. Each cadaveric forearm was incised along the midline of the hand, starting at the end of the forearm and extending to the base of the proximal phalanx of the middle finger. Additionally, a horizontal incision was made across the distal third of the palm. The dissection extended to Guyon's canal, which was fully exposed following the careful division of the transverse carpal ligament.

The BA was identified by tracing the neural branches emerging from the common palmar digital nerves (CDNs), specifically focusing on CDN3 and CDN4. Emphasis was placed on identifying and characterizing any communication between these branches originating from the MN and UN. Each instance of communication was measured using a digital caliper, including the distance from its origin in the palmar region to key anatomical landmarks.

To standardize dissection and measurement, vertical and horizontal distance reference points were used:

1. Vertical distance: measurements were taken from the bi-styloid line (a line between the radial and ulnar styloid) to the third metacarpophalangeal (MCP) joint. These reference points were based on established dissection techniques in prior anatomical studies, providing consistent orientation for analyzing the BA’s vertical position [1,7].

2. Horizontal distance: the horizontal distance was measured from the second to the fifth MCP joints, allowing for accurate mapping of the BA across the palmar region. This helped to define the BA’s horizontal location and its proximity to anatomical landmarks.

High-resolution images were captured at each dissection point to document the presence and characteristics of the BA. The images focused on nerve communications in the palmar region, specifically in the area between the third and fourth digits. Each cadaver’s BA was examined to assess the presence or absence of anastomosis, and the prevalence was calculated based on the data obtained. Five researchers participated in both data collection and analysis to ensure accuracy. To assess reliability, two separate researchers independently measured and typed each BA. If discrepancies in measurements occurred, the data points were averaged between the values obtained by each author, ensuring consistency in the results.

Prevalence rates for the BA were calculated, and the anatomical measurements of the BA (including distances from anatomical landmarks) were analyzed to determine the "probable zone" where the BA is most likely to occur.

## Results

In this study, the BA was identified in 83 of the 104 dissected hands from 26 males and 26 females, examined bilaterally, yielding a prevalence rate of 80%. The classification of neural patterns was first determined based on the anatomical location and number of their points of origin and insertion, followed by an analysis of the branching patterns within the BA itself. The origin of the BA was defined as the most proximal point of communication, while the insertion was defined as the most distal point.

**Figure 1:**
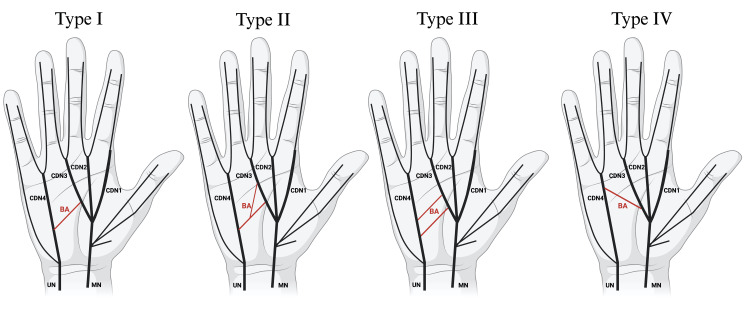
Diagrams from left to right represent type I (a single communication from UN to MN), type II (a bifurcating communication from UN to MN), type III (two communications from UN to MN), and type IV (a single communication from MN to UN). Type V could not be accurately illustrated in diagrammatic form. Image created in BioRender [13]. BA, Berrettini anastomosis; CDN1, common palmar digital nerve 1; CDN2, common palmar digital nerve 2; CDN3, common palmar digital nerve 3; CDN4, common palmar digital nerve 4; MN, median nerve; UN, ulnar nerve

To provide a comprehensive understanding of the observed anatomical variations, the patterns of the BA have been categorized and documented in Table 1, which is further supported by illustrative Figure 1. These diagrams depict the variations in origin, insertion, and branching of the BA, offering a visual reference for the anatomical configurations encountered.

**Table 1:** The BA’s categorical type along with their respective descriptions and prevalence. BA, Berrettini anastomosis; CDN3, common palmar digital nerve 3; CDN4, common palmar digital nerve 4; MN, median nerve; PDN, proper digital nerve; UN, ulnar nerve

BA Type	Description	Prevalence (%)
Type I	One origin from UN's CDN4 with one insertion on MN's CDN3	39/104 (38%)
Type IA	One origin from UN’s CDN4 and insertion on the MN’s medial PDN	4/104 (4%)
Type IB	One origin from UN's CDN4 with one insertion on MN's CDN3 with a branch extending to the cubital fossa	4/104 (4%)
Type IC	One origin from UN directly with one insertion on MN's CDN3	2/104 (2%)
Type ID	One origin from UN’s CDN4 with one insertion on MN directly	2/104 (2%)
Type II	One origin from UN's CDN4 with two insertions on MN's CDN3	14/104 (13%)
Type III	Two origins from UN's CDN4 with two insertions on MN's CDN3	8/104 (8%)
Type IV	One origin from MN's CDN3 with one insertion on UN's CDN4	8/104 (8%)
Type V	Complex and fibrous network between UN and MN	2/104 (2%)
-	-	Total prevalence = 83/104 (80%)

**Figure 2:**
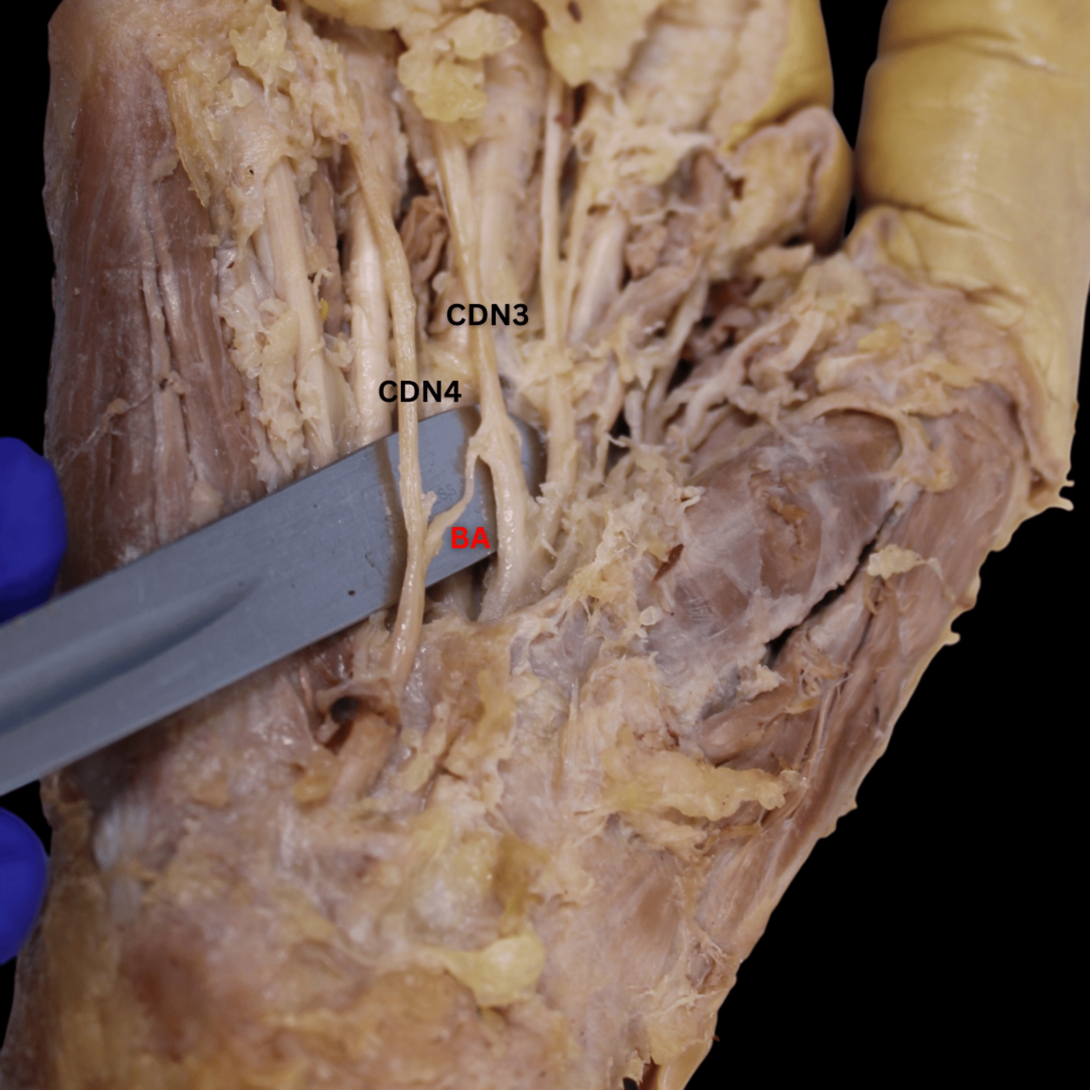
Cadaveric dissection reveals the BA’s type I variation, characterized by a single origin from the UN’s CDN4, which anastomoses onto the MN’s CDN3. BA, Berrettini anastomosis; CDN3, common palmar digital nerve 3; CDN4, common palmar digital nerve 4; MN, median nerve; UN, ulnar nerve

**Figure 3:**
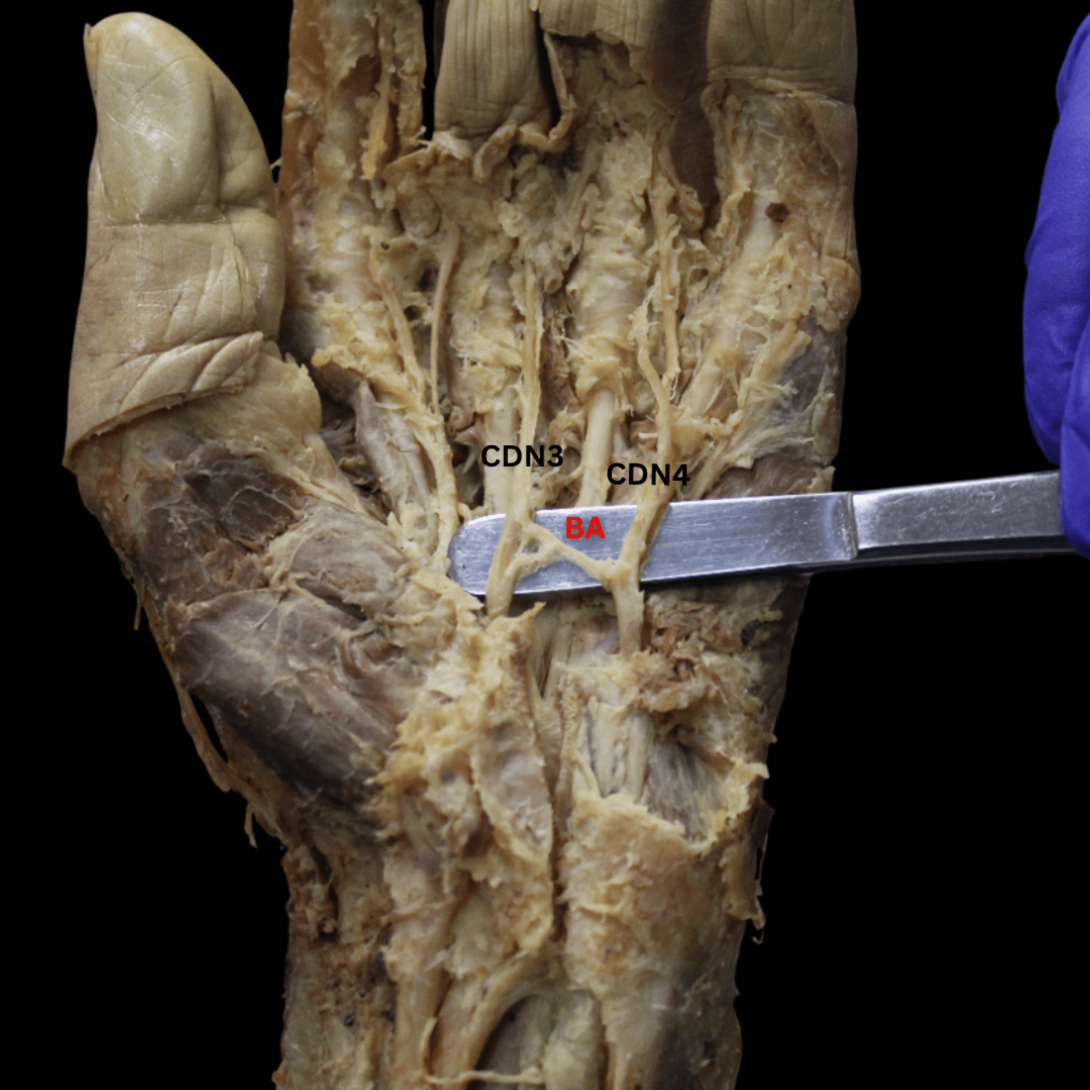
Cadaveric dissection reveals the BA’s type II variation, characterized by a single origin from the UN’s CDN4, which bifurcates and anastomoses twice onto the MN’s CDN3. BA, Berrettini anastomosis; CDN3, common palmar digital nerve 3; CDN4, common palmar digital nerve 4; MN, median nerve; UN, ulnar nerve

**Figure 4:**
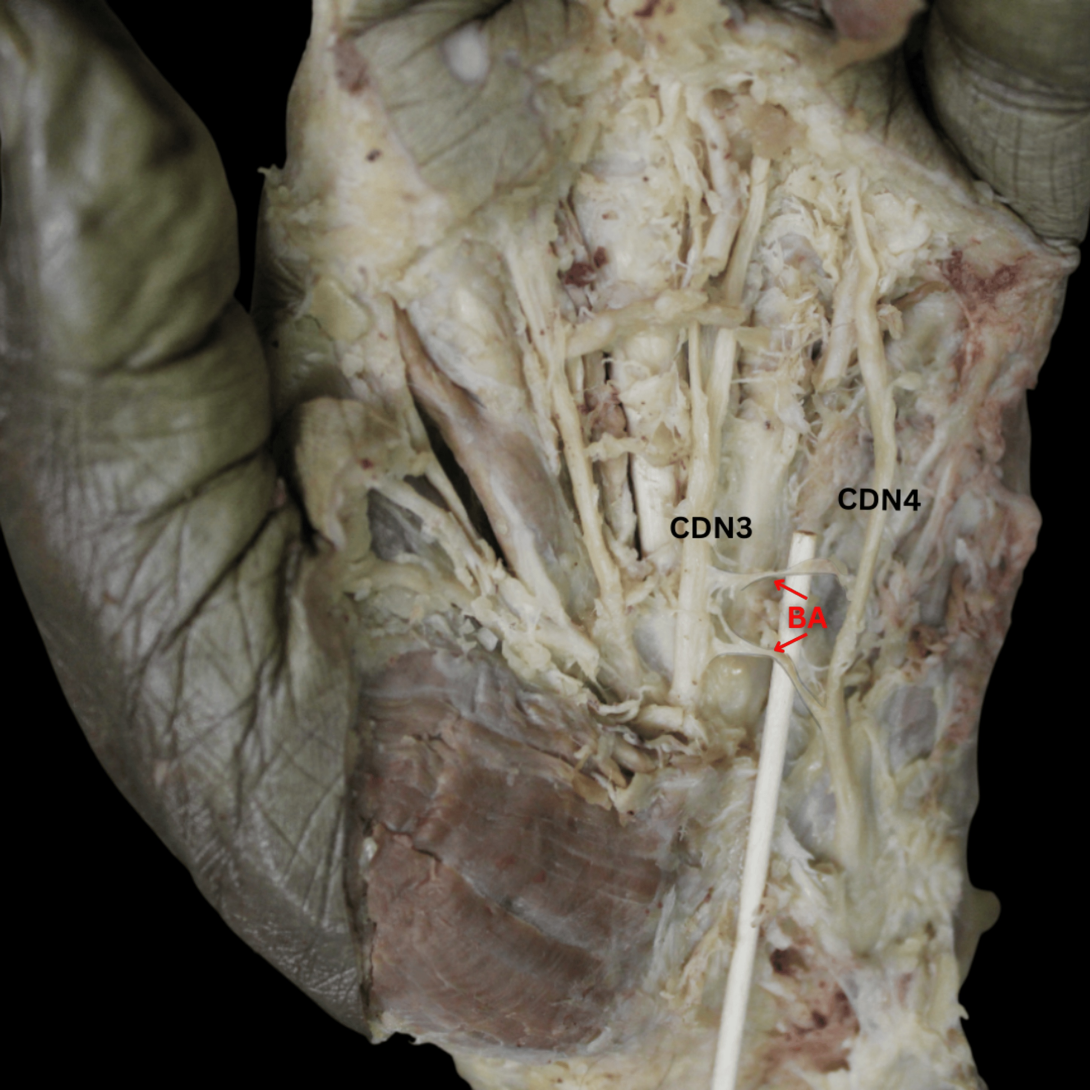
Cadaveric dissection reveals the BA’s type III variation, characterized by two origins from the UN’s CDN4, each inserting onto the MN’s CDN3. BA, Berrettini anastomosis; CDN3, common palmar digital nerve 3; CDN4, common palmar digital nerve 4; MN, median nerve; UN, ulnar nerve

**Figure 5:**
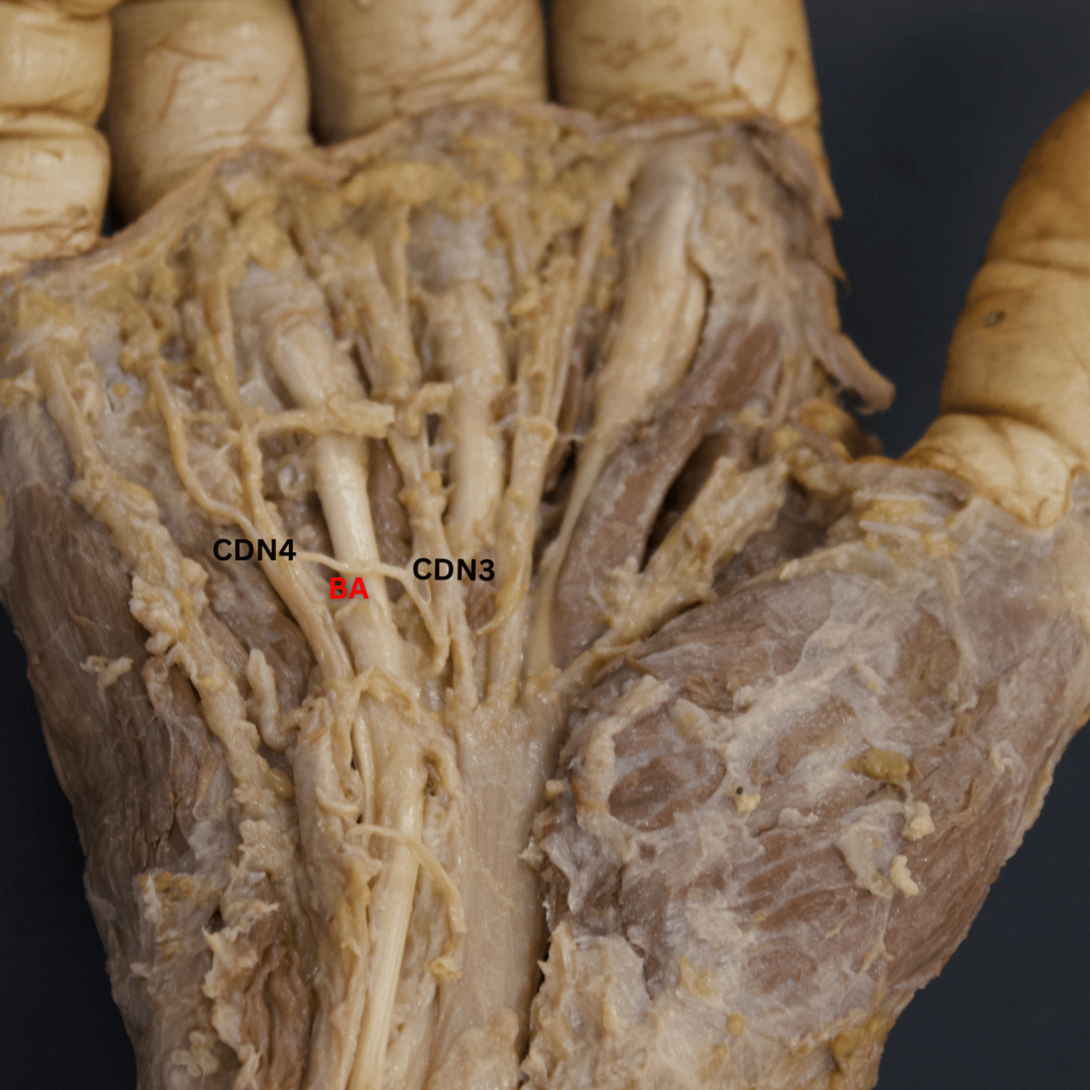
Cadaveric dissection reveals the BA’s type IV variation, characterized by a single origin from the MN’s CDN3, which inserts onto the UN’s CDN4. BA, Berrettini anastomosis; CDN3, common palmar digital nerve 3; CDN4, common palmar digital nerve 4; MN, median nerve; UN, ulnar nerve

**Figure 6:**
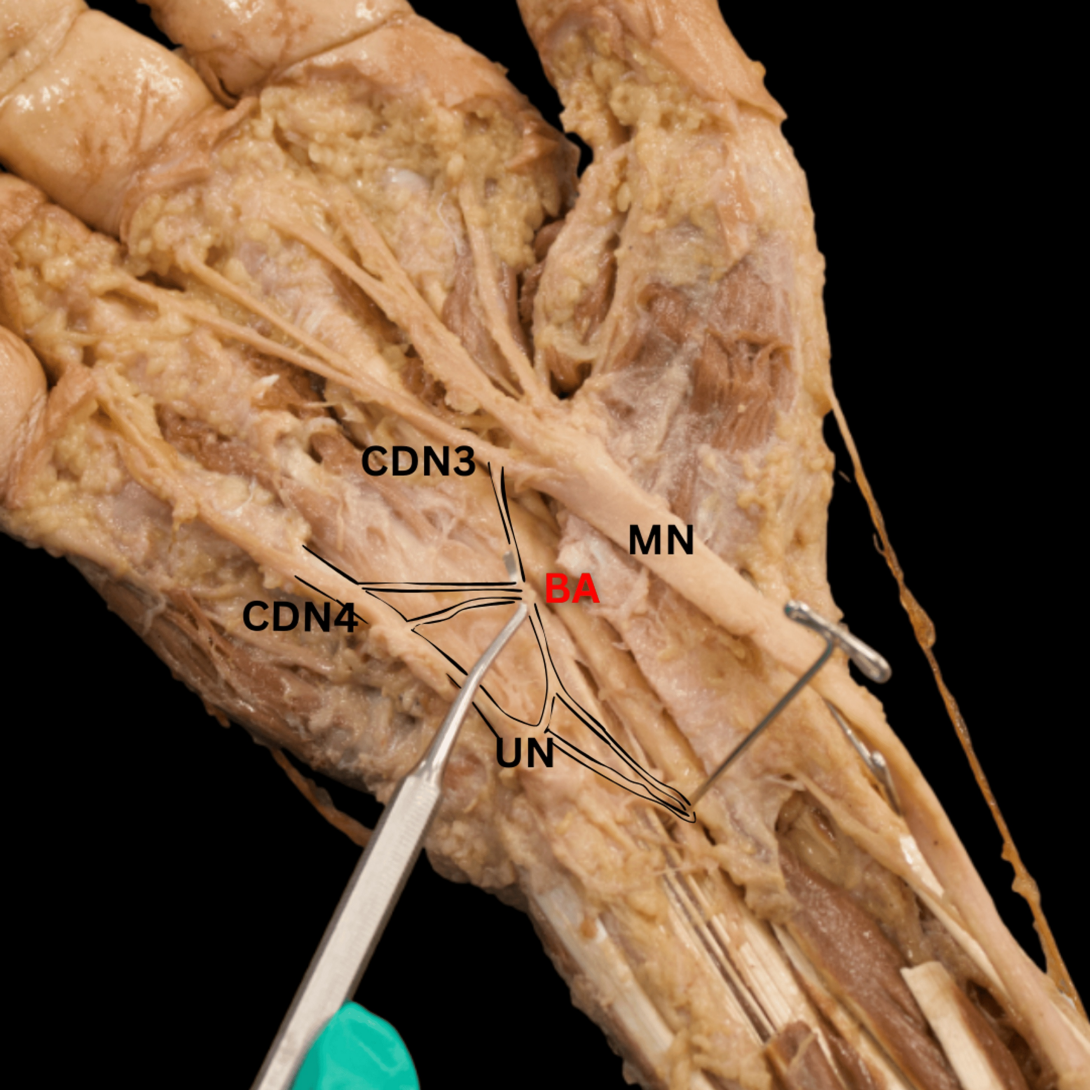
Cadaveric dissection reveals the BA’s type V variation, characterized by a complex network of multiple communications between the MN and UN. BA, Berrettini anastomosis; MN, median nerve; UN, ulnar nerve

Type I, the most common pattern, accounted for 38% of the cases and involved a single communication originating from the UN's CDN4 and inserting onto the MN's CDN3 (Figure 2). Several minor type I patterns, including types IA-ID, were characterized by a single origin on the UN and an insertion on the MN, either directly or through a derivative (CDN or proper digital nerve). These patterns collectively accounted for 12% of all cases. Type II, observed in 13% of cases, displayed a bifurcating pattern originating from CDN4 and inserting twice onto CDN3 (Figure 3). Type III, with two origins and two insertions, was found in 8% of cases (Figure 4). Type IV, a reverse configuration of type I, originated from the MN and inserted onto the UN, also comprising 8% of cases (Figure 5). Lastly, a complex, fibrous type V anastomosis, found in 2% of hands, was unique in its extensive network, particularly near the flexor retinaculum (Figure 6).

**Figure 7:**
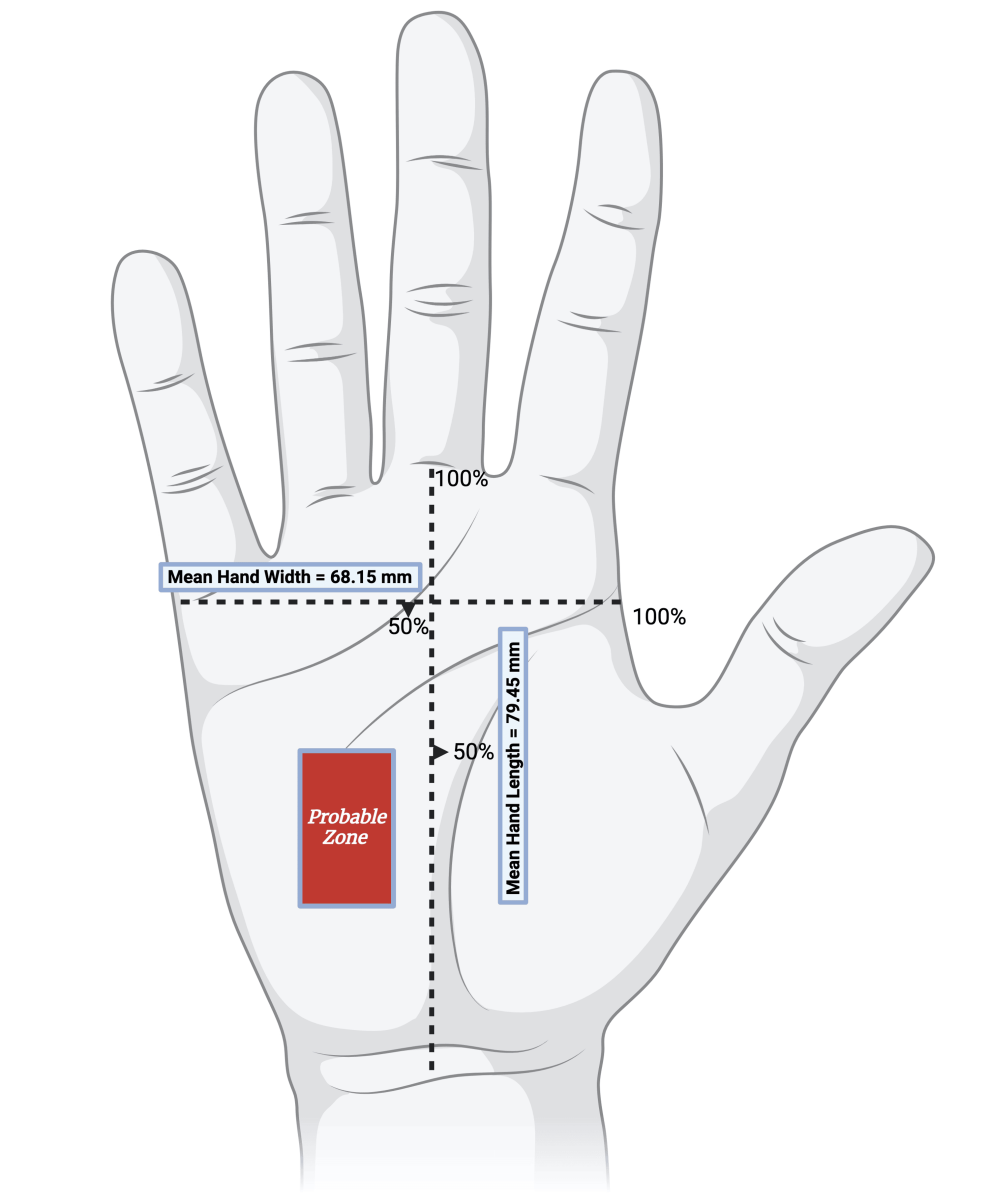
Diagram depicting the resultant probable zone of the BA within the hypothenar compartment of the hand. Image created in BioRender [14]. BA, Berrettini anastomosis

To further elucidate the anatomical mapping of the BA, we measured it in relation to other anatomical reference points to gain deeper insight into its location. The mean length of the communication was 22.03 ± 7.68 mm, with X and Y measurements of 12.34 ± 4.67 mm and 10.79 ± 5.61 mm, respectively. These were compared to the dimensions of the hand, including the horizontal plane (mean width of 68.15 ± 14.66 mm, measured from the second to the fifth MCPs) and the vertical plane (mean length of 79.45 ± 10.88 mm, measured from the bistyloid line to the third MCP joint). The combined dimensions of the hand and the BA created a rectangular area representing the average geography and location of the BA when these areas are superimposed (Figure 7). This area, referred to as the “probable zone,” is characterized by percentages of the hand's mean dimensions. In terms of the BA’s horizontal measurements relative to hand width, the probable zone extended from 26.11% to 44.21%. Similarly, by comparing the BA’s vertical measurements to the hand's length, the probable zone was found to range from 36.63% to 50.21% of the hand’s mean length.

## Discussion

The study provides valuable insights into the prevalence, classification, and clinical relevance of the BA in the hand. With a prevalence of 80%, the BA was found to be a common neural connection, and its variations in structure were higher than the pooled prevalence reported by Roy et al. (60.9%) but consistent with other reports with prevalence rates of 77.4% and 85% [1,2,15]. The classification system proposed here highlights both the common and rare patterns, adding clarity to earlier research. Type I, with its single connection between the UN and MN, was confirmed as the most prevalent form, aligning with findings in prior studies [1,2,9,12,15,16]. Notably, type II and type III patterns demonstrated variations in branching, with type II distinguished by a bifurcating connection and type III by dual origins and insertions. The rare type IV and type V anastomoses added complexity to the anatomical landscape, with the latter’s fibrous structure being particularly relevant in surgical contexts, as it may overlay critical areas such as the flexor retinaculum. Type V communication is not reported in the descriptions of the BA in many previous studies and is important to document, as it may be particularly vulnerable to iatrogenic injury. This classification highlights the overall fibrous nature of many BAs across all types, particularly near their origin and insertion points. This phenomenon has not been previously described in the literature on neural connections.

The introduction of the "probable zone" concept, a defined rectangular area where the BA is most likely to be found, enhances the precision of its anatomical mapping. This concept was developed by comparing the BA’s dimensions with the overall width and length of the hand, offering a more accurate reference for clinicians. Understanding the BA's position within this zone is clinically significant for procedures such as carpal tunnel release and surgeries involving neurovascular structures, where inadvertent damage to the BA could impair sensory function, particularly in the third and fourth digits. Other studies have previously identified "risk areas" and "high-risk surgical zones" and measured the BA to provide more insight into its geography [1,7,17]. However, most of the existing literature has measured only the proximal-to-distal length (Y-length) of the BA, without consideration of the medial-to-lateral length (X-length) in relation to the hand.

Despite the comprehensive nature of this study, several limitations should be addressed. The inability to image the BA before dissection might have led to the loss of fine neural branches, potentially affecting the accuracy of pattern identification. Future studies incorporating imaging techniques, such as MRI or ultrasound, could provide a non-invasive means of visualizing the BA in vivo, allowing for more detailed mapping. Additionally, the study underscores the need for standardized protocols in classifying BAs, as the variability in measurement techniques across studies limits the comparability of findings.

## Conclusions

The study confirms the high prevalence of the BA in human hands, with numerous anatomical variations in its communication patterns. These variations were consistently found within a defined "probable zone," where the BA is most likely to be located. Identifying this probable zone more precisely can be invaluable for surgical planning, particularly in procedures involving the hand and wrist. By recognizing the location and patterns of the BA, surgeons can minimize the risk of nerve damage, thereby preserving sensory and motor functions. Further investigation is recommended to refine this zone and enhance its clinical utility.
